# Supplementary material for: Graphene Klein tunnel transistors for high speed analog RF applications
Source: Sci Rep. 2017 Aug 29;7:9714. doi: 10.1038/s41598-017-10248-7 (PMC5575307; doi:10.1038/s41598-017-10248-7)
Supplement: Supplementary file 1 — Supplementary info [file 41598_2017_10248_MOESM1_ESM.pdf]

Supplementary Information
Graphene Klein tunnel transistors for high speed analog RF applications

Yaohua Tan[1,*,†], Mirza M. Elahi[1,†], Han-Yu Tsao[1], K. M. Masum Habib[1,2], N. Scott Barker[1], Avik W. Ghosh[1,§]
[1]Department of Electrical and Computer Engineering, University of Virginia, Charlottesville, Virginia 22904, USA
[2]Intel Corp., Santa Clara CA 95054, USA.
[*]yt5x@virginia.edu, [§]ag7rq@virginia.edu, [†]these authors contributed equally to this work

I. SELF-CONSISTENT POISSON ESTIMATION OF THE POTENTIAL PROFILE

As it is shown in Fig.1(e), we approximate the potential profile across the junction as a linear function.To support this approximation, we solved self-consistent Poission equations for the devices with backgate as shown in Fig.S1. We demonstrated this by solving the Poisson equation of the x-z plane cross section of the GKTFET as shown in Fig. S1(a). Here we have considered different backgate thickness and biasing conditions. The results of Possion calculation is shown by the following figure. It can be seen that the potential profile of both p-n, n-n and n^+-n junctions are approximately linear functions.

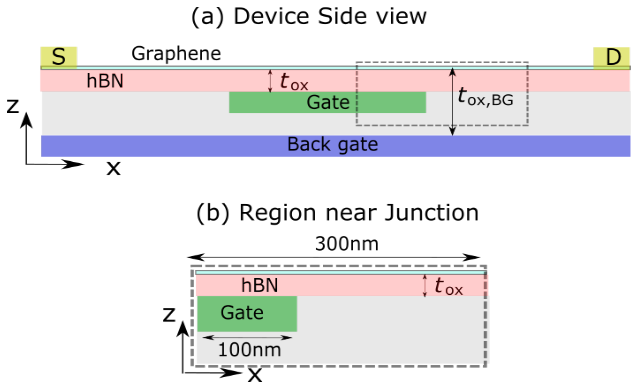

FIG. S1: (**a**) side view of the back gate GKTFET. (**b**) The region near the Junction. The potential contour in region (b) will be shown in Fig. S2.

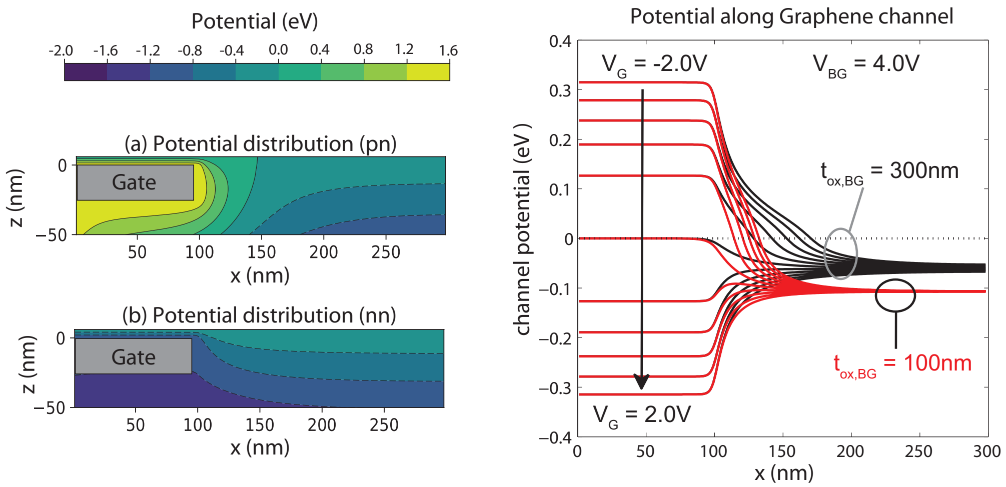

FIG. S2: The potential in graphene channel under different bias. The voltage contour of (**a**) p-n and (**b**) n-n cases, (**c**) potential distribution in the graphene channel for V_{BG} = 4.0 V (back-gate), and V_{GS}=-2.0 to 2.0 V (local gate). The t_{ox} for the gate (local) is 5 nm hBN. The t_{ox} for back-gate is 300 nm and 100 nm SiO_2. It can be seen that the potential changes approximately linearly from p (or n) to n^- region.
